# Supplementary material for: The Gut of Healthy Infants in the Community as a Reservoir of ESBL and Carbapenemase-Producing Bacteria
Source: Antibiotics (Basel). 2020 May 27;9(6):286. doi: 10.3390/antibiotics9060286 (PMC7345940; doi:10.3390/antibiotics9060286)
Supplement: Supplementary file 1 [file antibiotics-09-00286-s001.pdf]

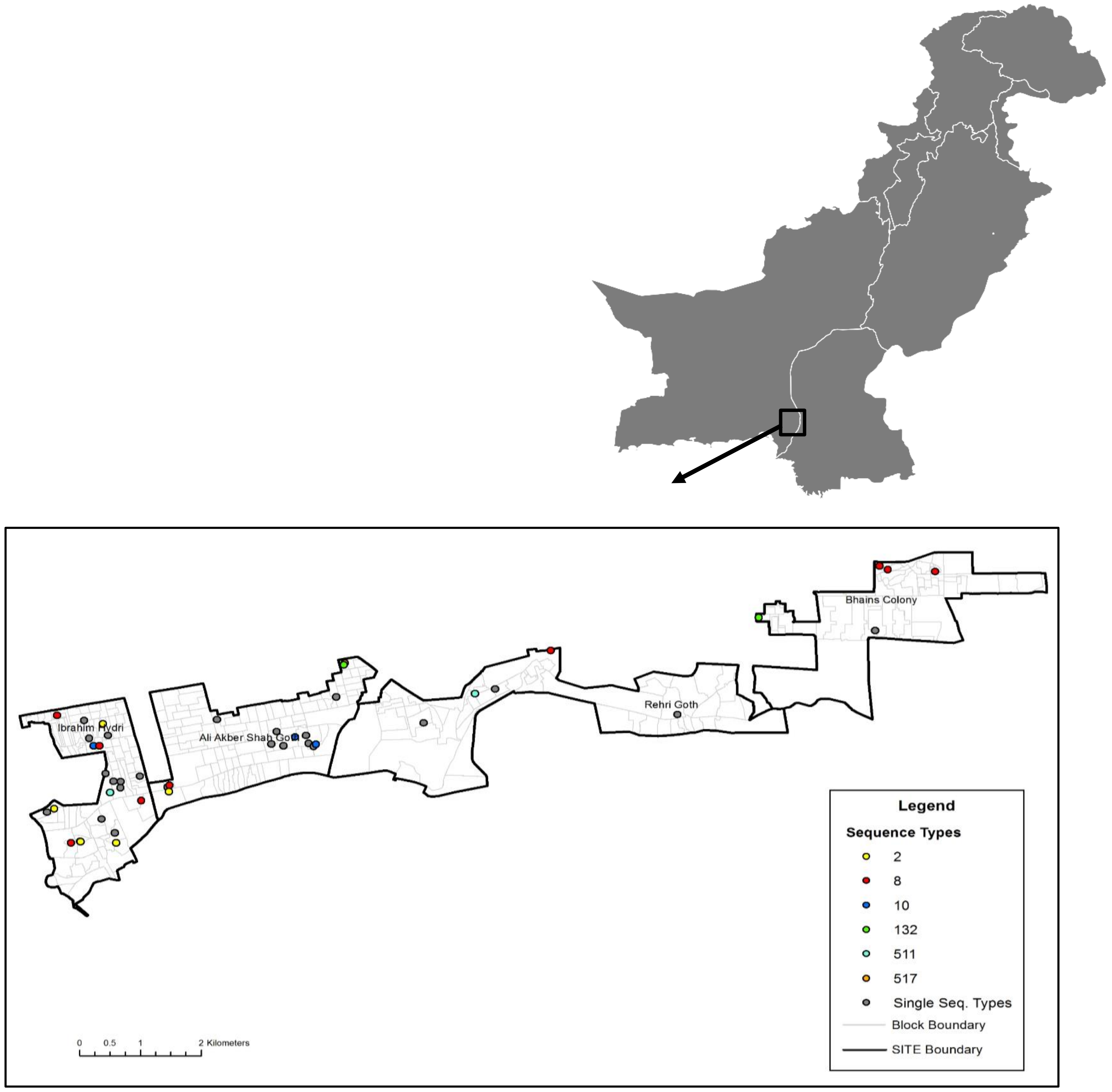

Supplemental figure 1: Distribution of the residences of infants who tested positive for ESBL *E. coli* enrolled at the four different study sites.
